# A Deep Learning-Based Camera Approach for Vital Sign Monitoring Using Thermography Images for ICU Patients

**DOI:** 10.3390/s21041495

**Published:** 2021-02-21

**Authors:** Simon Lyra, Leon Mayer, Liyang Ou, David Chen, Paddy Timms, Andrew Tay, Peter Y. Chan, Bergita Ganse, Steffen Leonhardt, Christoph Hoog Antink

**Affiliations:** 1 Medical Information Technology, Helmholtz Institute for Biomedical Engineering, RWTH Aachen University, 52074 Aachen, Germany; leon.mayer@rwth-aachen.de (L.M.); liyang.ou@rwth-aachen.de (L.O.); leonhardt@hia.rwth-aachen.de (S.L.); christoph.hoog_antink@tu-darmstadt.de (C.H.A.); 2 Eastern Health Clinical School, Monash University Melbourne, Box Hill, VIC 3128, Australia; dche0051@student.monash.edu (D.C.); ptimms@student.unimelb.edu.au (P.T.); ttay0005@student.monash.edu (A.T.); peter.chan@easternhealth.org.au (P.Y.C.); 3 Research Centre for Musculoskeletal Science and Sports Medicine, Manchester Metropolitan University, Manchester M1 5GD, UK; b.ganse@mmu.ac.uk; 4 Biomedical Engineering, Electrical Engineering and Information Technology, TU Darmstadt, 64289 Darmstadt, Germany

**Keywords:** camera-based vital sign measurement, infrared thermography, IRT, object detection, deep learning, optical flow, ICU monitoring

## Abstract

Infrared thermography for camera-based skin temperature measurement is increasingly used in medical practice, e.g., to detect fevers and infections, such as recently in the COVID-19 pandemic. This contactless method is a promising technology to continuously monitor the vital signs of patients in clinical environments. In this study, we investigated both skin temperature trend measurement and the extraction of respiration-related chest movements to determine the respiratory rate using low-cost hardware in combination with advanced algorithms. In addition, the frequency of medical examinations or visits to the patients was extracted. We implemented a deep learning-based algorithm for real-time vital sign extraction from thermography images. A clinical trial was conducted to record data from patients on an intensive care unit. The YOLOv4-Tiny object detector was applied to extract image regions containing vital signs (head and chest). The infrared frames were manually labeled for evaluation. Validation was performed on a hold-out test dataset of 6 patients and revealed good detector performance (0.75 intersection over union, 0.94 mean average precision). An optical flow algorithm was used to extract the respiratory rate from the chest region. The results show a mean absolute error of 2.69 bpm. We observed a computational performance of 47 fps on an NVIDIA Jetson Xavier NX module for YOLOv4-Tiny, which proves real-time capability on an embedded GPU system. In conclusion, the proposed method can perform real-time vital sign extraction on a low-cost system-on-module and may thus be a useful method for future contactless vital sign measurements.

## 1. Introduction

Intensive care units (ICUs) are among the most vital hospital wards, as they are reserved for patients with critical health conditions [1]. Here, continuous monitoring of vital signs is crucial for the early detection of an acute deterioration in health. The basic parameters monitored are heart rate (HR), blood pressure, respiratory rate (RR) and body temperature (BT), that provide information about the general physical status [2].

The monitoring of BT allows the observation of hypo- and hyperthermia, e.g., in inflammation. According to a study by Laupland et al., 16% of ICU patients have some type of hypothermia and up to 26% suffer from fever [3]. Erkens et al. observed dysregulation of BT in half of all patients in a German ICU. In general, BT is considered a significant predictor of mortality [4].

In addition, observations of changes in the respiratory rate can detect serious respiratory failure, which is the most common cause of admission to the ICU [5]. In 2015, 8% of all deaths in EU countries could be linked with respiratory diseases, which makes it the third main cause of mortality [6]. Despite continuous monitoring of respiratory activity in ICUs, the RR is the least accurately recorded vital sign in hospitals, despite its significance as a detector for early signs of deterioration [7]. Almost all sensors currently used for patient monitoring require direct contact to the body, but for a number of reasons, including handling and hygiene, contactless monitoring would be preferable. Moreover, the measurement quality of e.g., electrodes can vary with displacement. In the worst case, monitoring can cause medical adhesive-related skin injuries (MARSI) in patients with sensitive skin, such as infants or burn patients [8]. The replacement of disposable equipment (e.g., electrodes) is usually expensive and requires advanced medical knowledge for operation. Moreover, the environmental impact of medical waste production must not be underestimated.

To overcome the disadvantages of wired patient monitoring, contactless vital parameter acquisition has been investigated by research groups worldwide [9]. The development of camera-based techniques was initialized by Wu et al. in 2000, who used a CCD camera to extract dermal perfusion changes from the skin surface [10]. In addition to illumination-dependent camera technologies, Murthy et al. introduced infrared thermography (IRT) cameras in 2004 to extract the body surface temperature (BST) and RR from respiration-induced temperature changes in mouth and nose regions [11]. Subsequently, these techniques have seen great progress in accuracy and performance, due to improved computational efficiency and rapid developments in the field of machine vision. In this paper, a deep learning (DL)-based algorithm for the extraction of relative BST changes and RR from patients in the ICU using a low-resolution IRT camera is presented. A real-time object detection algorithm was used to extract signal-containing regions-of-interest (ROIs) in the frames. The head and chest regions were cropped to measure BST changes and breathing-related thorax movements from consecutive frames using an optical flow (OF) algorithm. Finally, a performance analysis was conducted to show real-time capability on embedded GPU modules for a low-cost implementation.

The further structure of this work is described as follows: Section 2 provides an overview of related works in the field of camera-based RR monitoring. Section 3 describes the dataset and the DL-based algorithm for vital sign extraction. Section 4 presents the performance results of the object detector and the contactless monitoring of RR and BST. Section 5 analyzes and reflects on the results of the presented approach. Finally, Section 6 summarizes the major findings and describes limitations of the algorithm.

## 2. Related Works

In the last decade, major advances have been developed in the field of camera-based vital sign monitoring. In 2011, Abbas et al. presented a method for respiratory monitoring from a tracked nose region in infrared images for neonates [12]. In the meantime, Lewis et al. worked on a similar tracking approach for adults to additionally estimate relative tidal volume changes [13]. In 2015, Pereira et al. presented an advanced approach to estimate RR from the nostrils using a high definition thermography camera [14]. Sun et al. measured RR and HR simultaneously from the face/nose region with a dual RGB/IRT camera system [15]. Elphick et al. conducted a larger study with more than 70 participants using a technique for facial analysis to track the nose region [16]. Although these methods showed high accuracy for the extraction of respiration, all approaches used highly expensive camera hardware and required a consistent line-of-sight to the nostrils, which restricts the position and angle of the camera. Furthermore, several tracking algorithms had to be applied offline after the actual recordings. Thus, no real-time capability existed.

Subsequently, computationally complex tracking algorithms were increasingly replaced by efficient DL-based methods. Real-time capable face and nose detectors (e.g., [17,18]) offer a great potential to enhance existing monitoring systems. In 2019, Kwasniewska et al. used neural networks in combination with low resolution thermography camera modules for a so-called super resolution approach to show feasibility of RR monitoring in nose-region images of only 80 × 60 px [19]. Furthermore, Jagadev et al. presented a machine learning-based measurement method where regression trees were used to track the nostrils [20]. The authors additionally investigated gradient techniques and support vector machines for ROI tracking [21]. Despite the high potential of machine vision in the field of thermography-based monitoring techniques, research is still at an early stage. Moreover, most groups worked on the extraction of respiration-related signals from the nasal region in thermography videos, which are, however, difficult to obtain in clinical environments. So far, the number of publications where IRT was used to monitor thorax movement for the extraction of RR is very limited. Nevertheless, studies were conducted in an animal trial with anesthetized pigs [22] and for RR monitoring of infants [23]. The application of DL methods for segmentation/detection in this context has not yet been covered in the literature. Finally, although commercial devices for medical thermography are available and used for e.g., tumor examinations, there is no approved IRT-based equipment for non-contact measurement of RR.

## 3. Materials and Methods

### 3.1. Experimental Setup and Dataset

**Figure 1:**
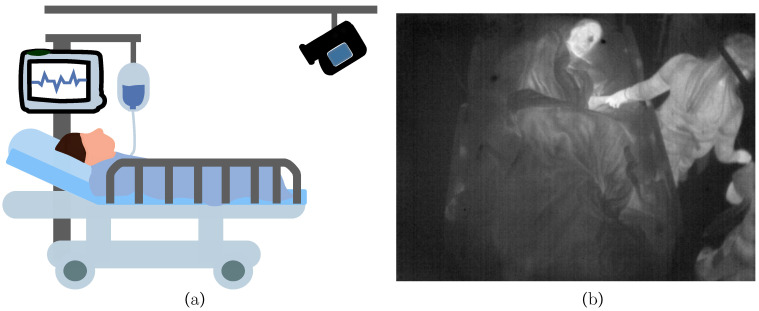
(**a**) Measurement setup in the intensive care unit (ICU) with an infrared camera attached to the ceiling. (**b**) Example infrared frame from patient.

The IRT datasets were recorded at the ICU of Box Hill Hospital in Melbourne, VIC, Australia, while the study was approved by the Human Research and Ethics Committee of Eastern Health, Melbourne, Australia (LR45-2017). Written informed consent was obtained from all patients. In total, 26 patients were recorded with the infrared camera Optris PI 450i (Optris GmbH, Berlin, Germany) at 4 frames per second (fps). The measurements were conducted with a spatial resolution of 382 × 288 px and a thermal sensitivity of 40 mK. In contrast to a previous study presented in 2019 [24], where the camera was mounted on a tripod and brought to the bedside, in the present study, the thermography device was attached to the ceiling. The patients were recorded for the length of their stay, which resulted in varying measurement durations. The measurement setup is depicted in Figure 1a.

The angle and distance of the camera were adjusted to optimize the field of view. This ensured an overall view of the patient and allowed the detection of clinical staff around the patient bed. An example infrared frame is illustrated in Figure 1b). No additional reference measurements were conducted in parallel to the camera recording to minimize disturbances in daily clinical procedures. Therefore, supplementary information about vital signs was only documented manually during routine examination.

All data of the 26 patients were analyzed. The training, validation and test datasets were created by randomly sampling 150 frames from every patient to train and validate the DL approach. This resulted in a subset of 3900 images. As shown in Table 1, 900 frames were sampled from six patients to form the test dataset, while data of the remaining 20 patients formed the training/validation set (3000 frames). This was done to prevent any training effect of the detector regarding the test dataset. Since reference data for RR were available from a patient monitor (Philips, Amsterdam, The Netherlands) using thoracic bioimpedence at hourly intervals just for six patients, these patients were defined as the test dataset with ground truth data. In total, 137 reference data points for RR were collected by the clinical staff and used to validate the OF method. As illustrated in Table 2, an additional subset of images was sampled from the patients of the test dataset by extracting 960 consecutive frames ( 240 s) before and after the clinical measurement procedure of RR (in overall 1920 frames or 8 ). These frames were extracted from the total IRT dataset.

**Figure 2:**
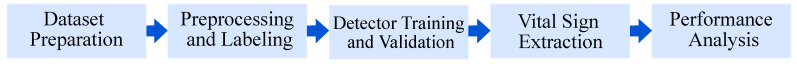
Overview of the algorithm.

Following datasets preparation, the frames were labeled and preprocessed for the subsequent steps of training and validation of the object detector (see Section 3.2 and Section 3.3). The trained model was then used to extract RR and BST from patients of the test dataset (Section 3.4). Finally, a performance analysis was conducted to show real-time feasibility on embedded GPUs. An overview of the algorithm is depicted in Figure 2.

### 3.2. Data Preprocessing

In a first step, all frames were normalized in terms of minimum and maximum temperature values, which was required for the detector training process and later application of the OF method. In consecutive frames with strongly changing temperature values (e.g., a hot drink was given to the patient), this technique resulted in different image contrasts. The normalization thus functions as an augmentation step to increase the diversity of the training set. Due to the standardized setup and the resulting small variability in camera perspective and distance, we assume that the training process would not benefit from additional, classical augmentation methods, such as rotation, flipping or scaling. This was already observed in previous publications [25]. Thus, no further augmentation strategies were investigated.

**Figure 3:**
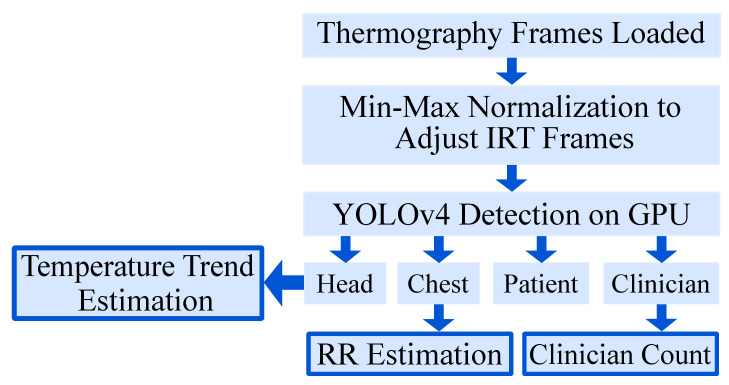
Overview of the segmentation algorithm.

**Figure 4:**
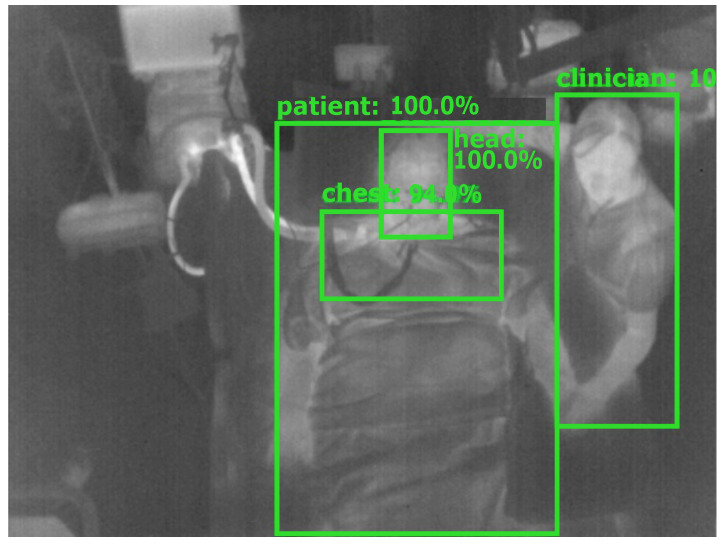
Detection result of YOLOv4.

The ground truth labeling was performed using the tool “Yolo_mark” [26]. In each frame, the following labels were applied (if available) with a bounding box: patient, patient chest, patient head and clinician. The first label contained information about the position of the patient to detect the presence and track global movement. In a next step, the head and the thorax were labeled for later vital sign extraction. Finally, a fourth label was introduced to distinguish between a patient and clinical staff or e.g., visitors and to quantify medical procedures or visits. Figure 3 gives an overview of the segmentation algorithm. In Figure 4, a detection result of the YOLOv4 algorithm is depicted.

### 3.3. Detector Training and Validation

In this work, the darknet implementation of the YOLOv4 object detection algorithm in Python by Bochkovskiy et al. [27] with the CSPDarknet53 backbone was employed. In comparison to the prior detector YOLOv3 of Redmon et al. [28], YOLOv4 outperforms YOLOv3 with respect to detection accuracy as well as speed, and allows the use of state-of-the-art GPU accelerated methods for training and inference. To further investigate the application of minimized network architectures on embedded GPU systems, the YOLOv4-Tiny was further evaluated. It has a smaller model size and faster inference speed, but it was unknown if it performed worse in terms of accuracy.

**Figure 5:**
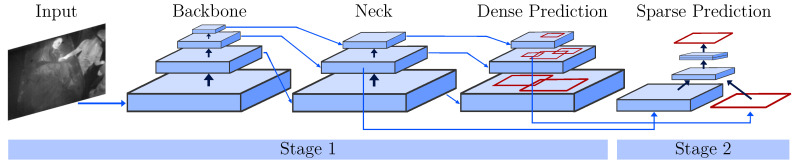
One- and two-stage object detectors modified from [27].

In contrast to two-stage object detectors like (fast) R-CNNs, the YOLO architecture is a one-stage detector. While region-based CNNs use a region proposal network to generate ROIs in a first stage and send the proposal to a pipeline for classification and regression, single-stage methods treat object detection as a “simple” regression problem (see Figure 5). Next to an input stage, a backbone and sequential neck and head (dense prediction) form the core of the YOLO detector. The backbone consists of pretrained feature extractors, which are fine-tuned using the detection dataset. In the neck, extra layers are used to extract feature maps of different stages of the backbone. The head is the main part responsible for the classification and regression of bounding boxes. To improve the detection accuracy, YOLOv4 uses two methods: Bag-of-Freebies and Bag-of-Specials. While the former describes techniques including (mosaic) data augmentation, CutMix or DropOut, the latter performs methods including max-pooling and a novel mish activation. Please refer to [27] for further information about the network architecture.

**Figure 6:**
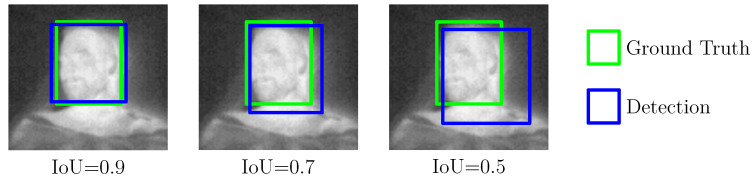
Intersection over Union as an evaluation metric for object detectors.

For model training, a high-performance desktop computer was used, running Ubuntu 18.04 and featuring an Intel Xeon Gold 6128 processor, two NVIDIA Quadro RTX5000, and 400 GB RAM. To accelerate the training process, both GPUs were deployed in combination with CUDA 10.2, cuDNN 7.6.5, and OpenCV 4.2.0. The training process can be described as follows: before the actual training step, the configuration files for both detector models were adapted for the specific characteristics of the dataset and number of labels (see [29]). Then, the model weights were trained first on one GPU for 1000 iterations with an image size of 416 × 416 px. Afterwards, another 7000 iterations were performed on both GPUs by using the partially trained model. Note that this technique was proposed by Bochkovskiy to conduct a more stable, yet accelerated training [29]. Early stopping was used to prevent overfitting by evaluating the inference results on the validation set. A 10-fold cross-validation (CV) was performed to measure the model performance and obtain estimates of the generalization process during the training step. The results will be presented in Section 4.1. During the CV, the intersection over union (IoU), mean average precision (mAP) and the $F_{1}$ score were analyzed with an intersection/detection threshold of 0.5 as common evaluation metrics for object detectors. While the IoU is a measure of the overlap between the bounding boxes from detection and ground truth, the mAP score specifies the accuracy of the detector’s predictions over all classes. Figure 6 illustrates an example of three different IoU results for the head of a patient. Furthermore, the $F_{1}$ score describes the harmonic mean of precision and sensitivity (true positive rate) of the validation process.

### 3.4. Vital Sign Extraction

The segmented IRT data were used to quantify disturbances due to medical procedures or visits, to estimate a temperature trend, and to estimate the RR.

#### 3.4.1. Quantification of Disturbances

To quantify the occurrence of medical procedures or visits to the patient, the presence of the bounding box “clinician” was evaluated in a binary fashion.

#### 3.4.2. Temperature Trend Estimation

The bounding box for the head was used to crop the facial part for temperature extraction. To generate an estimate of the BST, the maximum temperature value of the bounding box was selected.

Note that although the thermal sensitivity of the used camera is 40 mK, the absolute accuracy only amounts to ±2 ${}^{\circ }C$. Thus, in our approach, we computed a temperature trend by using the relative deviation from the temperature value of the first frame, as fluctuations in the range of the absolute accuracy could lead to incorrect classifications regarding hypo-/hyperthermia. To reduce the influence of camera drift, the room temperature was estimated from a 5 × 5 px ROI in the upper left corner of the IRT video and used for compensation.

#### 3.4.3. Respiratory Rate Estimation

**Figure 7:**
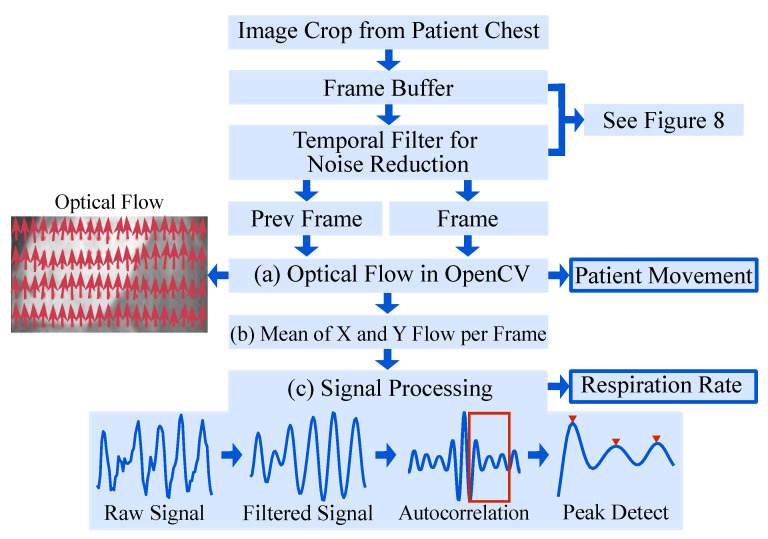
Overview of the algorithm for RR extraction.

The proposed approach makes use of the fact that respiration causes subtle motions of the chest of the patient which can be quantified from IRT images via optical flow. An overview of the algorithm is given in Figure 7.

The OpenCV implementation of the OF algorithm by Farnebäck et al. [30] was chosen. The initial step is to approximate each neighborhood of two consecutive frames by quadratic polynomials, to receive global displacement information. Subsequently, the global polynomial is replaced with local approximations by a polynomial expansion. This results in a spatially varying displacement field, which contains the movement of pixels. For more information on the OF algorithm, the interested reader is referred to [30].

**Figure 8:**
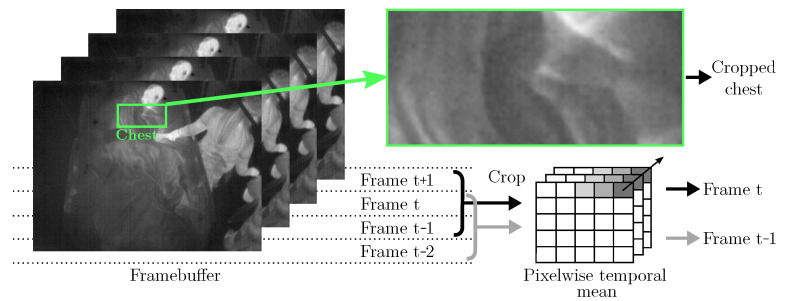
Temporal filtering as preprocessing for Farnebäck’s OF algorithm.

It is known that the signal-to-noise ratio and the contrast of IRT images are generally lower compared to other (visual) camera modalities, which complicates image processing. Especially, the application of OF using unfiltered IRT images may not be feasible, as these methods are based directly on the intensity levels of pixels. Therefore, a temporal filter algorithm was implemented, see Figure 8: The cropped chests from four consecutive frames were buffered. Pixel-wise mean operations on the first three frames (t−2, t−1, t) were used to generate the first input (“Prev Frame” in Figure 7), while the last three frames (t−1, t, t + 1) were used to generate the second input to the OF algorithm (“Frame” in Figure 7).

The OF algorithm (Figure 7a) returned the displacement field, i.e., the motion of each pixel in the chest region as visualized by red arrows in “Optical Flow”, Figure 7. All displacement vectors were spatially averaged (Figure 7b) to extract the mean motion of the chest. This “Raw Signal” (Figure 7c) was filtered using a 2nd-order Butterworth bandpass filter with breathing-related cutoff frequencies of 0.15 Hz and 0.44 Hz. Next, the autocorrelation of the filtered signal was computed to quantify the signal’s self-similarity. Finally, the largest peak in the range of the respiratory rate was selected to calculate the respiratory rate.

### 3.5. Real-Time Feasibility on Embedded GPUs

The low-cost system-on-modules Jetson AGX Xavier (approx. 700$, November 2020) and the less performant version Jetson Xavier NX (approx. 400$, November 2020) (NVIDIA, Santa Clara, USA) were used for inference to show the feasibility of applying the trained detector in combination with embedded GPU systems. Both development boards provide a 64-bit CPU, a NVIDIA Volta GPU, including tensor cores, 16 GB (AGX Xavier) and 8 GB (Xavier NX) of RAM, and a dual DL accelerator for optimized inference. These modules combine high performance and power efficiency in a miniaturized form factor to deliver the power of accelerated DL to embedded systems. Low-cost portable devices can be implemented for real-time camera-based monitoring systems by using these systems. Both modules were selected for the inference of YOLOv4 and YOLOv4-Tiny to determine the performance and usability for real-time monitoring of vital signs. The results of a detailed performance analysis will be provided in Section 4.1.

## 4. Results

### 4.1. Detector Performance

A patient-wise 10-fold CV was performed to quantify the overall model performance and obtain reliable estimates of the generalization process during the training step. In each iteration, data from two patients of the training set were defined as validation set in every fold, while data from the other 18 patients were used as training data. In Table 3, the results of the CV for both detector architectures, YOLOv4 and YOLOv4-Tiny, are presented. Furthermore, the evaluation metrics for the final prediction of the hold-out test dataset are presented, where all data of the CV were used for training.

**Figure 9:**
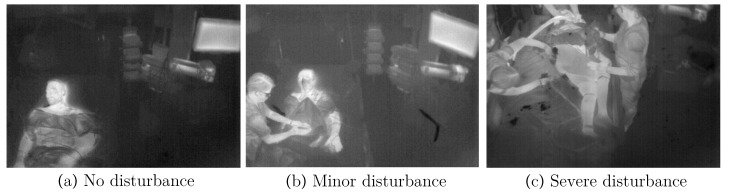
Different levels of disturbances in IRT frames.

In general, the results showed mAPs of 0.95 (YOLOv4) and 0.94 (YOLOv4-Tiny), respectively $F_{1}$ scores of 0.91 and 0.93 for the test dataset. In addition, IoUs of 0.7 for the larger model and 0.75 for the tiny version were observed. The evaluation metrics did not show a standard deviation (SD) higher than 0.08 for the individual folds. This indicates a generalization of the model, so overfitting was prevented. Furthermore, the results provided evidence that the performance on the test dataset did not vary from the training dataset. Nevertheless, the detector performed worse on folds 7 and 10 due to differences between the measurement conditions (e.g., ceiling height or frequency of medical interventions). Different levels of disturbances due to medical interventions can be observed in Figure 9. While optimal conditions can be seen on the left side, Figure 9b,c show minor and severe disruption.

In addition to the averaged results from the 10-fold CV, Table 4 presents the detection performance (averaged IoU and the averaged precision) for all individual classes. While there was negligible difference in the overall performance of the detectors, both YOLOv4 and the tiny model showed a reduced accuracy for the class chest. Nevertheless, the precision for the classes indicated an adequate result for thorax recognition, which was used for further processing.

Both detection models were used for inference on embedded GPU systems to show the feasibility of real-time performance on low-cost system on modules. In Table 5 the mean performance on the GPU platforms introduced in Section 3.5 are presented in fps. The trained network models were transferred to the Jetson development kits and evaluated on the test dataset. Even the cheapest module Xavier NX had real-time capabilities with 47 fps for the YOLOv4-Tiny detector. Furthermore, the YOLOv4 detector showed a performance of 9 fps on the same GPU. Since our study was conducted with a temporal resolution of 4 fps, both detectors can be used to implement real-time vital sign monitoring on a Jetson module.

A further analysis of the real-time feasibility of the entire algorithm gave no limitations regarding the application of the approach on embedded GPU systems. Although the OF implementation of OpenCV is CPU-based, it has not led to any restrictions of the real-time capability, even on the less-performant Jetson systems. Since the cropped chest ROI had an average resolution of only 100 × 40 px, the computational costs had an appropriate dimension for the multi-core CPUs. Furthermore, the performance of the OF approach was in the range of several hundred fps, so that the influence on the total runtime is negligible.

### 4.2. Temperature Trend Estimation

**Figure 10:**
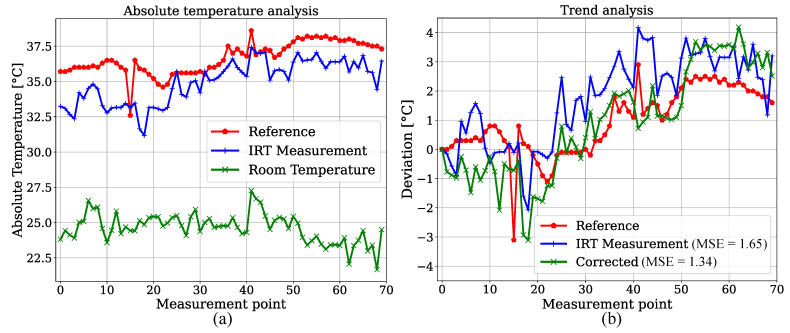
(**a**) Absolute temperature analysis for reference measurements in bladder. (**b**) Relative deviations for room temperature correction.

We created an estimator for BST by measuring relative head temperature deviations in the IRT frames. Since reference data for BST were not provided for the patients in the test dataset, 70 datapoints of one participant of the training set with corresponding temperature values measured in the bladder were extracted and compared with IRT. This was done to show feasibility for the temperature trend estimations presented later. The YOLOv4-Tiny was used due to the similar results for both detectors. In order to exclude a training effect in the detection step, the model weights from a fold were used, in which this specific patient was not part of the training dataset. An analysis of absolute temperatures can be observed in Figure 10a. As expected, an underestimation of the bladder reference is revealed. Next to the reference and camera-based measurements, an estimation of the room temperature is shown as described above. A trend analysis was performed by calculating the deviations from the initial measurement point. Figure 10b shows that the IRT trend overestimated the reference deviations. If the estimated room temperature is subtracted for correction, the MSE of the estimated trend decreases from 1.65 K to 1.34 K.

**Figure 11:**
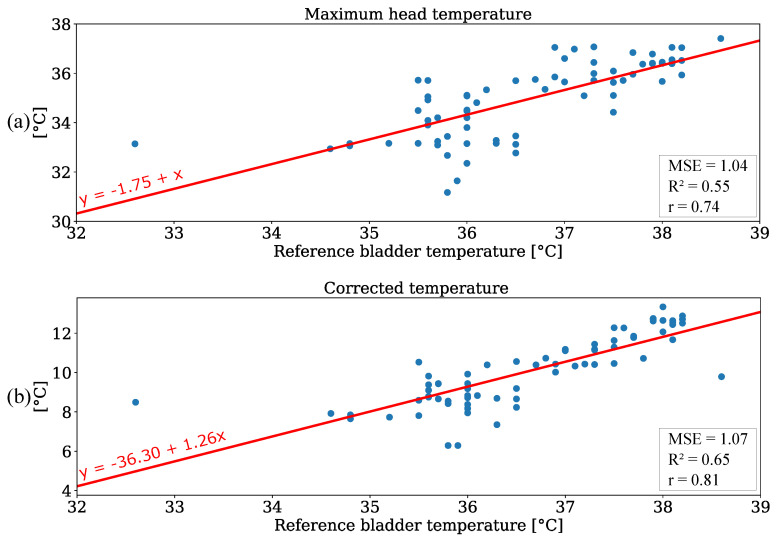
Regression analysis for (**a**) maximum head temperature and (**b**) corrected temperature values.

Linear regression was carried out to investigate the accuracy of IRT measurements. Since the reference data were limited to 70 temperature values from one patient, the focus of this analysis was to emphasize the linear correlation of both variables rather than to train a regression model for prediction. The results are depicted in Figure 11a. The regression resulted in a slope of 1 and an intercept of −1.75. A MSE of 1.04 and a $R^{2}$ of 0.55 were observed. These results indicate a positive correlation between the bladder temperature and IRT measurement. An additional analysis was performed for the ambient temperature correction. In Figure 11b the absolute values of IRT measurements were adjusted using the room temperature as depicted in Figure 10a. This resulted in a slight increase of the MSE (due to outliers) and a higher coefficient of determination for the regression. Furthermore, Table 6 shows the results for an analysis without the outliers at reference temperatures of 32.6 ${}^{\circ }C$ (unphysiological temperature) and 38.6 ${}^{\circ }C$ (corrupted ambient correction). The evaluation metrics are provided for both unedited and corrected temperature values. Here, an MSE of 0.72 and a $R^{2}$ of 0.77 were observed for the corrected values. These results demonstrate that an IRT camera can be used to perform thermal state measurements by extracting the maximum temperature value from the head of a patient.

**Figure 12:**
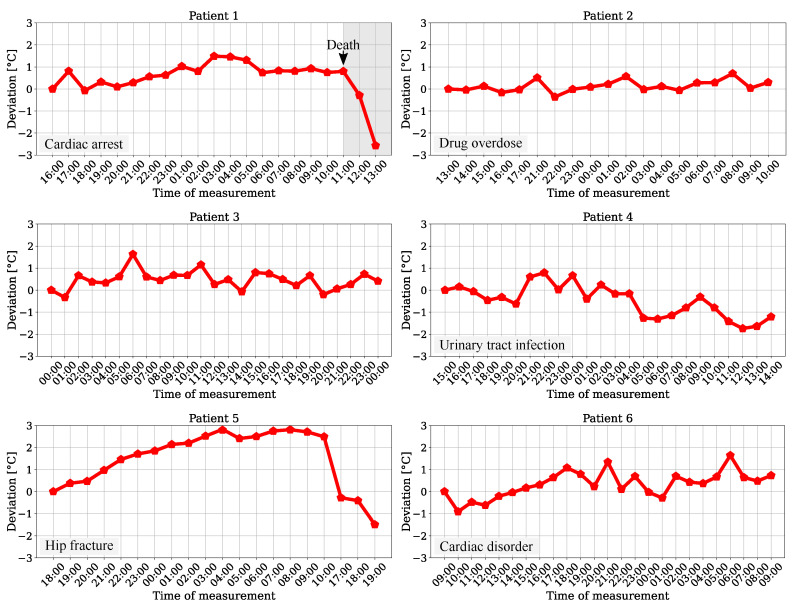
Head surface temperature deviation for patients from the test dataset.

The temperature trend for the six patients of the test dataset was determined by using the averaged maximum values of the head surface temperature of all frames from a measurement point (240 frames). The results are presented in Figure 12.

Since the patients were recorded for the length of their stay in the ICU, the plots show hourly, chronological measurements for temperature variations. While a slight rise can be obtained for the trend of patient 6, there was a long-term drop in temperature drift for patient 4. These progressions could indicate a pathological instability (deviation > 1 ${}^{\circ }C$) and indicate hypothermia or hyperthermia. The results for patients 2 and 3 showed no clear ascending or descending trend, but were in a range of approx. 1 ${}^{\circ }C$, which is outside physiological normothermal variations of 0.5 ${}^{\circ }C$ [31]. An analysis for patients 1 and 5 revealed significant deviations, which could indicate a pathological condition of the patient. Especially for patient 1, who passed during the measurement at 11 AM, a drop in temperature was recorded after death. Due to the ambient correction step and the dimensions of the temperature deviations, which showed strongly different behavior, the impact of possible sensor drifts of the camera during measurement as cause for the relative changes can be excluded. Furthermore, the results of a former reference data analysis showed the feasibility of a continuous and contactless temperature trend measurement for a monitoring system in the ICU using an IRT camera.

### 4.3. Extraction of Respiration Rate

**Figure 13:**
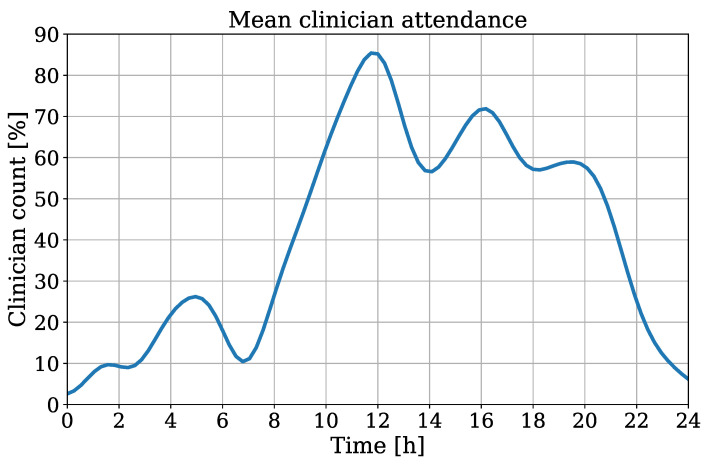
Interpolated clinician attendance from all patients of the test dataset.

Besides the temperature trend, the algorithm was used to extract the RR using movements of the thorax which were derived from an OF implementation. The attendance of clinical staff/guests was detected to quantify the incidence of medical examinations/visits. This information could be used to determine a quality index for RR extraction. In case of an overlap between the ROIs of the chest and clinician, the crop used as input for the OF algorithm could contain non-patient movement, which would interfere with the respiration signal. In Figure 13 the interpolated clinician attendance is illustrated for all patients of the test dataset.

Here, a count of 100% indicates the continuous presence of medical staff/visitors during the recording. The timestamps of all measurements were chronologically sorted for an interpolation of all extracted clinician counts. This results in an averaged attendance over one day. A coherence between daytime and clinician presence can be obtained, which indicates an obvious decrease of attendance in night hours.

**Figure 14:**
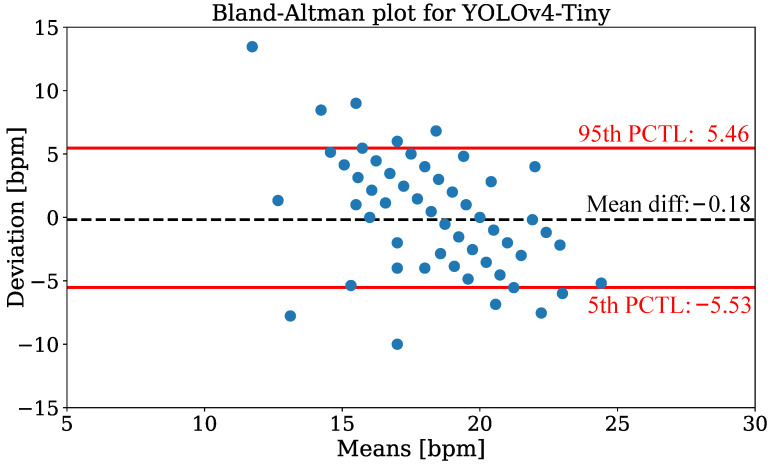
Bland-Altman plot of respiration extraction for the YOLOv4-Tiny detector.

In Figure 14, the Bland-Altman plot using YOLOv4-Tiny is depicted to compare the camera-based RR measurement technique with annotated reference data from all patients of the test dataset. The data were evaluated for discrete measurement times where respiration reference was provided by clinical annotation. The plot shows the RR deviation against the means on the *x*-axis. The analysis revealed a mean difference of −0.18 and percentiles of 5.46 (95th), respectively −5.53 (5th). While the estimation is virtually unbiased in terms of the mean difference, the error exhibits a negative correlation, i.e., larger values are under-estimated while small values are over-estimated. In Table 7 the mean absolute errors (MAEs) for RR extraction are presented. The evaluation showed mean MAEs of 2.79 bpm (YOLOv4) and 2.69 bpm (YOLOv4-Tiny).

**Figure 15:**
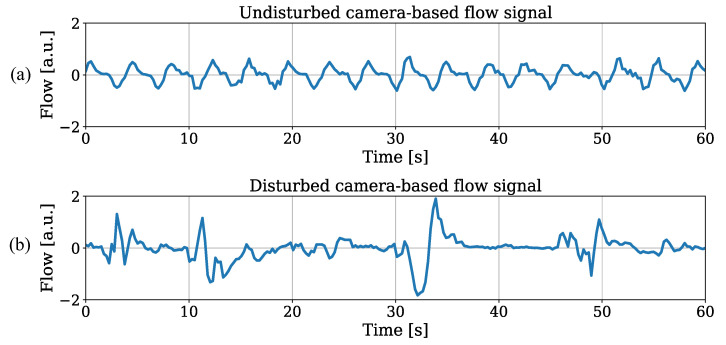
(**a**) Low disturbance level and (**b**) high disturbance level in raw flow signals.

Since similar results for both trained object detectors were observed, the illustration of RR estimation for the tiny model was sufficient. Besides outliers with strong deviations, the results revealed promising outcomes for IRT-based RR extraction from thorax movement. An individual analysis showed MAEs in the range of 0.67 bpm (patient 1) and 4.78 bpm (patient 3). While the extraction performed well on patients 1 and 2, the deviations were increased for the other recordings. The utmost accuracy in patient 1 can be explained by continuous sedation and constant mechanical ventilation during the entire stay on ICU without any disruptive factors. These optimal factors for measuring respiration-induced movements were not given in any other patient of the dataset. In Figure 15a, an undisturbed raw flow signal extracted from OF of patient 1 is illustrated. An example for a disturbed flow signal is depicted in Figure 15b, where an exact extraction of the respiratory rate is much more difficult.

Due to fails in the detection step of the algorithm, which can be explained with severe disturbances during recording, 26 measurement points were excluded from the analysis (111 of 137 measurement points left). The coverage of the detection is presented in Table 8. While the relatively lower coverage for patient 2 could be explained with recurrent seizure events (where OF-based RR measurement was excluded), the results for the other patients were due to detection fails of the chest region at the beginning of the video. This happened when severe disturbances were present in the first frames of the IRT measurement.

## 5. Discussion

### 5.1. Detector Analysis

The DL-based approach YOLOv4 was applied for the initial detection step of the algorithm. Although the relatively low number of labeled images used for training and validation could explain drawbacks in later detection performance, the presented metrics showed promising results for all classes in the hold-out test dataset. While the fact that results for the tiny version were expected to be worse due to the reduced model structure, a comparison of both detection models showed a similar performance. The evaluation metrics of the label chest showed even better performance for the tiny model. This could be due to the effect of model pruning, which allows reducing the number of unnecessary parameters and layer connections for the tiny detector, while accuracy remains stable and inference is accelerated. Nevertheless, the relatively lower IoU of the thorax could be explained by the level of complexity of detecting the chest. While the other classes always show similar shapes or contours (e.g., head), strongly changing contrasts between foreground and background (patient, clinician) and strong anomalies in the image (clinician), the thorax is located in the upper third of the patient and has less features to extract.

While a classification of the results for the labels patient, chest, and clinician is difficult due to a lack of similar detection problems in the literature, the performance of head detection can be compared to results for RGB images. El Ahmar et al. implemented real-time capable CNNs to detect a head ROI and shoulder keypoints in RGB-Depth images [32]. The authors achieved an averaged IoU of 0.69 for the label head using approx. 1500 images for training and 162 frames for testing. Saqib et al. compared different DL frameworks for head detection in RGB images from the HollywoodHeads dataset and observed an mAP of 0.791 for the VGG16 CNN architecture [33]. The good performance for head detection of the trained YOLOv4 models (test dataset: IoU: 0.84, mAP: 0.99) could be explained by differences between RGB and grayscale IRT images. Due to the fact that warm objects are obviously highlighted in the thermal frame, the complexity of detection could be decreased.

### 5.2. Temperature Extraction

The results for BST measurements revealed the potential of an implementation of the system in a clinical environment for monitoring using a low-cost IRT camera. Although a precise extraction of absolute BST using camera-based techniques still remains a challenge, we have shown high correlation coefficients for recorded (corrected) face temperatures and in a reference dataset (r = 0.75 with outliers). The application of a correction step to compensate room temperature effects resulted in similar correlation outcomes compared to the literature (r = 0.79), even though our camera distance was at least twice as large [34]. Nevertheless, no reference data were available for the patients from the test dataset, so the extracted, plausible temperature trends have to be evaluated in future work.

### 5.3. Monitoring of Respiration Rate

In contrast to the methods already presented in the literature, where temperature changes in the nasal region were used for RR extraction (e.g., [11,16]), our approach is based on quantifying respiration-related movements of the thorax using IRT. Despite the fact that the published approaches often had higher accuracies, many methods were conducted in a controlled laboratory study and depended on a line-of-sight to the nostrils for signal extraction, whereas our real-time OF method only needs a low-resolution ROI of the chest. While published techniques based on nasal regions resulted in a MAE of 0.33 bpm (controlled study) [14] or a mean bias of 0.67 bpm (clinical study) [24], a movement tracking algorithm in a patient study showed an averaged MAE of 2.07 bpm [23]. This analysis emphasizes the difficulty of the RR estimation for recordings in clinical environments. The latter technique based on respiration-induced movement shows worse performance compared to the laboratory studies and was in the same range as our OF approach. Nevertheless, the limitation of the required camera position to record a close-up of the nose region complicates a possible application of the technique in daily clinical practice. The presented recordings were disturbed by numerous movement artifacts, e.g., during mealtime, medical procedures or medical conditions such as seizures. Especially such conditions make an accurate extraction of the RR very difficult. Since movement artifacts generate disturbances in a large frequency band, which includes the physiological breathing frequency, today an adequate noise compensation is still the main challenge in camera-based vital sign measurements. Despite the challenges, the low-cost approach has great potential to be used as a continuous, contactless monitoring of the RR in clinical environments. Especially the measurement during night hours showed promising results.

## 6. Conclusions and Outlook

In this paper, we presented an approach for DL-based real-time extraction of vital signs using contactless IRT. A dataset of 26 patients recorded in an ICU was used to train and validate the object detectors YOLOv4 and YOLOv4-Tiny. A 10-fold CV was performed to quantify the overall detection performance. It has shown promising results for robust detection of the trained labels. While an IoU of 0.70 was observed for the YOLOv4 model on the test dataset, the tiny model showed a superior IoU of 0.75. The BST trend was measured by detecting the head and RR was extracted by using an OF algorithm looking for chest movements. A corrected regression analysis for the trend analysis resulted in an MSE of 0.72 ${}^{\circ }C$. The RR extraction showed MAEs of 2.79 bpm (YOLOv4) and 2.69 bpm (YOLOv4-Tiny).

While the extraction of temperature trends from the relative changes of head surface temperature showed the potential of detecting and tracking pathological changes, the comparison of the extracted RR with reference revealed several challenges. Unfortunately, movement disturbances complicated the camera-based extraction of RR. Nevertheless, during the night and for patients with low movement artifacts, the algorithms showed promising results. In future work, more IRT recordings with additional reference data for BT should be analyzed. We assume that larger datasets for training could improve the overall results of the YOLOv4 detection models. The RR extraction could be improved using the tracking information of clinical staff/visitors. The corruption of raw motion signals due to overlaps with the patient ROI could be avoided by neglecting these signal components in advance. We are confident that the use of a low-cost IRT camera system in combination with DL algorithms on an embedded GPU module could contribute to a reduction of wired sensor technologies for patient monitoring in ICUs and enhance the use of unobtrusive real-time capable vital sign acquisition.

## Figures and Tables

**Table 1:** Dataset division for training and validation of YOLOv4.

Dataset	Training/Validation	Test
Patients	20	6
Frames	3000	900
RR Reference	0	137

**Table 2:** Dataset sampling for evaluation of the OF algorithm.

Dataset	OF Evaluation
Patients	6
Frames	1920 per RR Reference

**Table 3:** Results of a patient-wise 10-fold CV.

	YOLOv4			YOLOv4-Tiny		
**Fold**	**IoU** ${}_{0.5}$	**mAP** ${}_{0.5}$	**F** ${}_{1}$	${\mathrm{IoU}}_{0.5}$	**mAP** ${}_{0.5}$	**F** ${}_{1}$
1	0.72	0.99	0.92	0.81	0.99	0.99
2	0.76	0.98	0.94	0.82	0.98	0.95
3	0.72	0.90	0.94	0.73	0.88	0.93
4	0.80	0.74	0.97	0.83	0.74	0.98
5	0.71	0.99	0.92	0.76	0.98	0.98
6	0.81	0.97	0.98	0.79	0.97	0.99
7	0.65	0.84	0.86	0.67	0.86	0.89
8	0.72	0.97	0.92	0.79	0.98	0.97
9	0.75	0.95	0.93	0.75	0.93	0.92
10	0.69	0.88	0.97	0.64	0.85	0.81
Mean	0.73	0.92	0.93	0.76	0.92	0.93
SD	0.05	0.08	0.04	0.06	0.08	0.07
Test	0.70	0.95	0.91	0.75	0.94	0.93

**Table 4:** Quantitative detection results on the test dataset (900 images).

	avg IoU [%]				mAP [%]			
**Detector**	**Head**	**Patient**	**Chest**	**Clinician**	**Head**	**Patient**	**Chest**	**Clinician**
YOLOv4	84	76	54	70	99	99	87	91
YOLOv4-Tiny	82	78	66	70	99	98	87	89

**Table 5:** Mean performance on different GPU platforms for the test dataset.

Platform	YOLOv4 [fps]	YOLOv4-Tiny [fps]
Jetson Xavier NX	9	47
Jetson AGX Xavier	19	86
Quadro RTX5000	80	296

**Table 6:** Regression results without outliers.

	w/o Correction	Correction
MSE	0.98	0.72
$R^{2}$	0.56	0.77
r	0.75	0.88

**Table 7:** Mean absolute errors for RR extraction in bpm.

		Patient						Mean
		1	2	3	4	5	6	
MAE	YOLOv4	0.67	1.64	4.78	2.26	3.69	3.72	2.79
	YOLOv4-Tiny	0.67	1.64	4.71	2.25	3.69	3.18	2.69

**Table 8:** Coverage of successful detections.

	Patient					
	1	2	3	4	5	6
Coverage [%]	86	60	88	95	84	78
